# Cognitive enhancement with Salience Network electrical stimulation is influenced by network structural connectivity

**DOI:** 10.1016/j.neuroimage.2018.10.069

**Published:** 2019-01-15

**Authors:** Lucia M. Li, Ines R. Violante, Rob Leech, Adam Hampshire, Alexander Opitz, David McArthur, David W. Carmichael, David J. Sharp

**Affiliations:** a Computational, Cognitive and Clinical Imaging Lab, Division of Brain Sciences, Department of Medicine, Imperial College London, W12 0NN, UK; b Department of Psychology, University of Surrey, GU2 7XH, UK; c Centre for Neuroimaging Science, Denmark Hill, SE5 8AF, UK; d Department of Biomedical Engineering, University of Minnesota, Minneapolis, MN, 55455, USA; e David Geffen School of Medicine, UCLA, Los Angeles, CA, 90095, USA; f Biomedical Engineering Department, Kings College London, SE1 7EP, UK

**Keywords:** Salience Network, Stop Signal Task, Transcranial direct current stimulation, TDCS, Response inhibition, Cognitive control, White matter tract, Fractional anisotropy, Functional MRI

## Abstract

The Salience Network (SN) and its interactions are important for cognitive control. We have previously shown that structural damage to the SN is associated with abnormal functional connectivity between the SN and Default Mode Network (DMN), abnormal DMN deactivation, and impaired response inhibition, which is an important aspect of cognitive control. This suggests that stimulating the SN might enhance cognitive control. Here, we tested whether non-invasive transcranial direct current stimulation (TDCS) could be used to modulate activity within the SN and enhance cognitive control. TDCS was applied to the right inferior frontal gyrus/anterior insula cortex during performance of the Stop Signal Task (SST) and concurrent functional (f)MRI. Anodal TDCS improved response inhibition. Furthermore, stratification of participants based on SN structural connectivity showed that it was an important influence on both behavioural and physiological responses to anodal TDCS. Participants with high fractional anisotropy within the SN showed improved SST performance and increased activation of the SN with anodal TDCS, whilst those with low fractional anisotropy within the SN did not. Cathodal stimulation of the SN produced activation of the right caudate, an effect which was not modulated by SN structural connectivity. Our results show that stimulation targeted to the SN can improve response inhibition, supporting the causal influence of this network on cognitive control and confirming it as a target to produce cognitive enhancement. Our results also highlight the importance of structural connectivity as a modulator of network to TDCS, which should guide the design and interpretation of future stimulation studies.

## 1 Introduction

Response inhibition is an important aspect of cognitive control, and can be assessed with the Stop Signal Task (SST). During SST performance, participants must inhibit an automatic motor response when an infrequent ‘stop’ signal appears. The Salience Network (SN) has an important role in cognition, particularly in tasks involving attending to and responding to unexpected but salient stimuli (Sridharan et al., 2008; Menon and Uddin, 2010). When inhibiting a response during the SST, brain activity is characterised by SN activation and concurrent Default Mode Network (DMN) deactivation (Sharp et al., 2010; Li et al., 2006; Hampshire and Sharp, 2015; Touroutoglou et al., 2012). This pattern of anti-correlated activity relates to SST performance, with greater SN activation and linked DMN deactivation associated with better performance and disruption of this pattern associated with poor performance (Congdon et al., 2011; Bonnelle et al., 2012; Weissman et al., 2006).

The Salience Network may be functionally segregated into dorsal and ventral components that support cognitive and emotional/affective control respectively (Touroutoglou et al., 2012). The key SN regions activated during SST performance comprise the dorsal components: the dorsal anterior cingulate cortex/presupplementary motor area (dACC/preSMA) and the right anterior insula (Touroutoglou et al., 2012; Seeley et al., 2007). The structure of the white matter tract connecting the rAI and the dACC/preSMA strongly predicts DMN deactivation during response inhibition, which is in turn related to SST performance (Bonnelle et al., 2012). Therefore, the SN is a promising target for brain stimulation aimed at modulating cognitive control and other cognitive networks, and structural connectivity within the SN might influence any physiological and behavioural effects attributable to stimulation. The right inferior frontal gyrus (rIFG), overlying the rAI, exhibits high intrinsic functional connectivity with the rAI (Touroutoglou et al., 2012), and frequently co-activates with the rAI during tasks, such as the SST, involving attentional control and response inhibition (Hampshire and Sharp, 2015; Aron and Poldrack, 2006; Aron et al., 2014; Hampshire et al., 2010). This makes the rIFG the ideal superficial cortical target for modulating SN function.

Transcranial direct current stimulation (TDCS) delivers weak electrical currents to the brain through scalp electrodes, and modulates neuronal excitability underneath the electrode (Stagg and Nitsche, 2013; Purpura and Mcmurtry, 1965). Its ability to modulate cognitive function has been widely investigated in both clinical and non-clinical work (Kuo and Nitsche, 2012). However, group level effects are inconsistent, with high inter-individual variability in the behavioural response to TDCS, leading to scepticism about its effects on the brain (Horvath et al., 2014, 2015).

A major issue is that there is limited understanding of how TDCS affects the brain networks important for cognitive control. There has also been little systematic exploration of the factors influencing the behavioural response to TDCS (Li et al., 2015). Individual variability in structural connectivity within stimulated networks has previously been shown to influence behavioural and physiological response to TDCS, but these studies did not investigate complex cognitive tasks (Rosso et al., 2014; Bradnam et al., 2012; Lin et al., 2017). The differential effects of anodal and cathodal TDCS are also not fully understood, particularly at the level of the neuronal circuit, brain network and behaviour. Opposing effects of anodal and cathodal TDCS on one level may be accompanied by similar effects of the two polarities on another level. In vitro studies suggest that anodal TDCS increases and cathodal TDCS inhibits neuronal excitability (Stagg and Nitsche, 2013; Purpura and Mcmurtry, 1965). However, the anodal-excitatory/cathodal-inhibitory dichotomy is often not seen at the behavioural level, suggesting a more complex interaction of polarity and brain activity, particularly for cognitive control (Jacobson et al., 2012).

One approach to understanding the effects of TDCS on cognition is to use functional (f)MRI to investigate the effects of TDCS on cognitive brain networks. Studies combining fMRI and TDCS have found that TDCS, both anodal and cathodal, to a single region can produce changes in activation and functional connectivity beyond the local stimulation region (Sehm et al., 2012a, 2013; Amadi et al., 2014; Hone-Blanchet et al., 2015; Polanía et al., 2012; Polania et al., 2012; Antal et al., 2011; Peña-Gómez et al., 2012; Sankarasubramanian et al., 2017; Park et al., 2013; Callan et al., 2016). However, most of these studies did not acquire fMRI simultaneous to TDCS, only investigated resting state fMRI or stimulated primary sensorimotor regions. Therefore, it is difficult to extrapolate from these results to an understanding of the effects of TDCS on brain networks subserving cognitive control.

Here we investigated the physiological and behavioural effects of stimulating the SN with TDCS. We concurrently acquired fMRI and delivered TDCS to the rIFG/rAI, whilst healthy participants performed the SST. We tested the hypotheses that rIFG/AI TDCS: 1) with anodal TDCS improves SST performance; 2) that variability in SN structure, measured using diffusion tensor imaging, influences the response to TDCS; 3) that rIFG/AI TDCS modulates remote brain activity (i.e. regions not directly within the electrical field), and 4) that there are distinct, but not necessarily opposing, effects of anodal and cathodal TDCS on network function and behaviour.

## 2 Materials and methods

### 2.1 Participants

We recruited healthy volunteers from the Imperial College Clinical Research Facility healthy volunteers list, with no history of neurological or psychiatric illness (n = 26, 13F:13M) (mean age 38 years, s.d. 15.5 years). All volunteers gave written informed consent. The study conforms to the Declaration of Helsinki and ethical approval was granted through the local ethics board (NRES Committee London – West London & GTAC). All participants were naïve to TDCS. Two participants were excluded from behavioural analyses due to problems acquiring response data, and two further participants were excluded from DTI analyses as this was not acquired.

### 2.2 Experimental protocol and Stop Signal Task

**Fig. 1:**
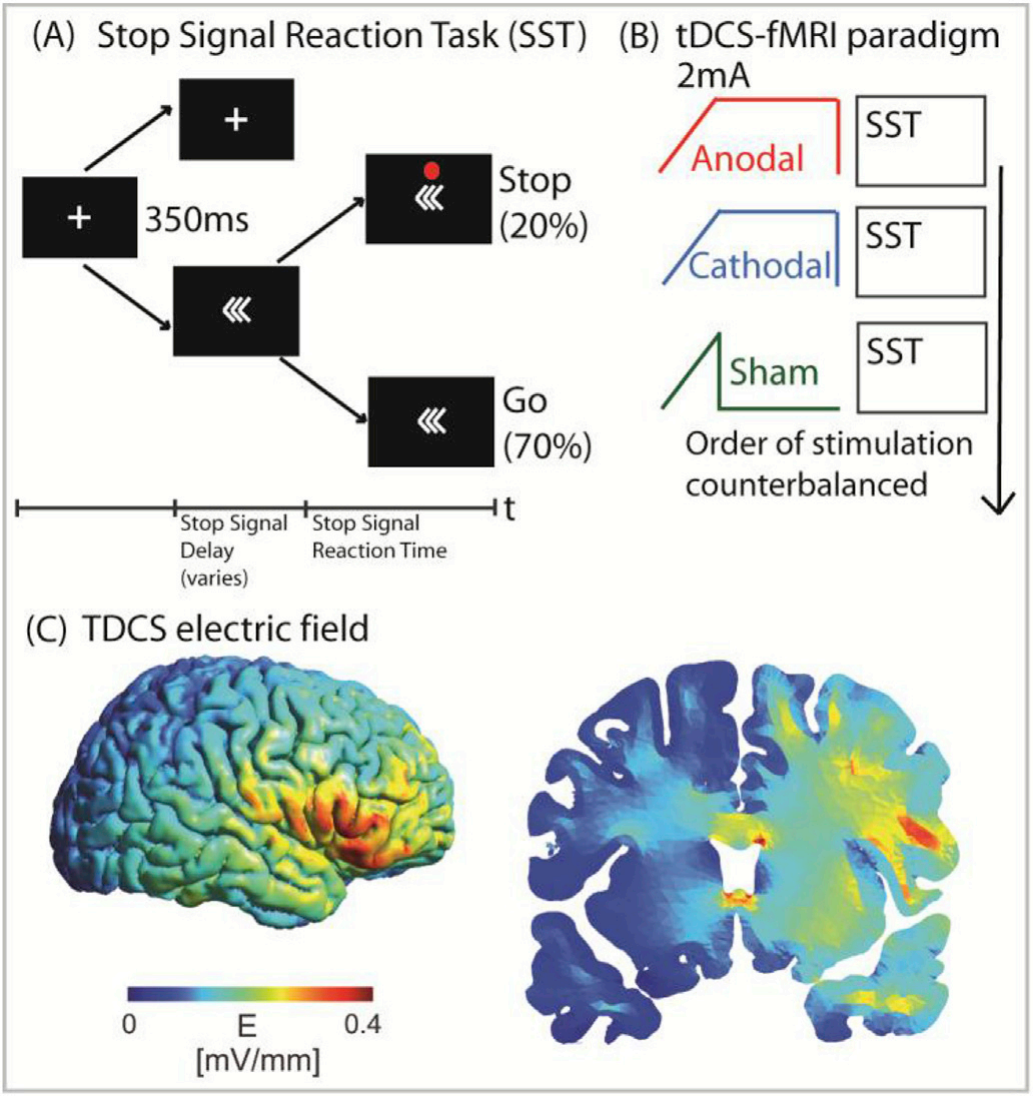
(A) Stimuli in the Stop Signal Task (SST). (B) The TDCS/fMRI paradigm, comprising 3 runs each of the SST with concurrent TDCS (anodal, cathodal and sham), with a 2–3 min break between each run. (C) Modelling showing maximum electric field strength over the rIFG, with current spreading into the underlying right anterior insula, as well as the white matter tracts.

Functional MRI was acquired whilst participants performed the Stop Signal Task (SST), in an event-related design (Fig. 1), the details of which have been previously described (Sharp et al., 2010; Bonnelle et al., 2012). In brief, participants are instructed to press a button held in their left or right hand in response to left or right pointing arrows respectively (the ‘go'signal). Infrequently, a red dot, the ‘stop’ signal, appeared above the arrow after a variable interval. Participants had to withhold their button press in response to the ‘stop’ signal. There were 184 trials in total, comprising 20% ‘stop’ trials, 70% normal (‘go’) trials and 10% rest trials, lasting 4mins 12secs in total. To minimise tactical waiting for the appearance of the ‘stop’ signal, a negative feedback screen saying “Speed Up” was presented if slowing reaction times were detected (Bonnelle et al., 2012). The task was programmed in Matlab (Mathworks, Natick, MA) using Psychtoolbox (Brainard, 1997) and responses were recorded through a fiberoptic response box (NordicNeuroLab, Norway), interfaced with the stimulus presentation PC.

Each participant performed 3 runs of the SST, under sham, anodal and cathodal TDCS. The runs were sequential but with brief 2–3 min break in between each run during which the participant remained in the MRI scanner. After each run of TDCS-fMRI, participants were asked if they thought they had stimulation or not, and to rate (from 1 to 5) the sensations (itching, pain, metallic taste, burning, anxiety, anything else) they felt during the run. The order was counterbalanced between participants to minimise risk of systematic bias from any potential carryover effects.

### 2.3 Analysis of behavioural results

The main outcome measure was the stop signal reaction time (SSRT). This is a composite measure that accounts for an individual's motor reaction time. It is calculated as [Mean reaction time – Stop Signal Delay], where stop signal delay (SSD) is the interval between presentation of the arrows and appearance of the red dot ‘stop’ signal which produced successful stopping on 50% of ‘stop’ trials. A low SSRT indicates good task performance. We also extracted the Stop Incorrect RT, which is the mean reaction time for ‘stop’ trials where the participant did not manage to inhibit their response. Statistical analyses of task performance during active (anodal or sham) compared to sham stimulation, using student's ttests of paired samples to assess effect of active compared to sham stimulation, were conducted using Matlab (Mathworks, Natick, MA) and R (www.r-project.org). Bonferroni correction was used to correct for multiple comparisons across different tracts and stimulation conditions when investigating the effect of white matter FA on behavioural response to stimulation.

### 2.4 fMRI acquisition

T1 and functional MRI (fMRI) sequences were acquired on a 3T Siemens Verio (Siemens, Erlangen, Germany), using a 32-channel head coil. FMRI images were obtained using a T2*-weighted gradient-echo, echoplanar imaging (EPI) sequence, 3mm3 isotropic voxel, repetition time (TR) 2 s, echo time (TE) 30 ms, flip angle (FA) 80°, field of view 192 × 192 × 105 mm, 64 × 64 matrix, 35 slices, GRAPPA acceleration factor = 2. Standard T1-weighted structural images were acquired using an MP-RAGE sequence, 1 mm^3^ isotropic voxel, TR 2.3 s, TE 2.98 ms, inversion time 900 ms, FA 9°, field of view 256 × 256 mm, 256 × 256 matrix, 160 slices, GRAPPA acceleration factor = 2.

### 2.5 Delivery of transcranial direct current stimulation

TDCS was delivered concurrently to SST task performance and fMRI acquisition using a MR-compatible battery-driven stimulator (NeuroConn GmbH, Ilmenau, Germany), with a previously described circuit (Violante et al., 2017). The ‘active’ electrode (4.5 cm diameter circular rubber electrode) was placed over F8 (based on the 10–20 EEG International system), corresponding to the pars triangularis of the rIFG, and the ‘return’ electrode (7 × 5cm rectangular rubber electrode) was on the right shoulder with its longitudinal axis parallel to the coronal plane (centre of electrode placed over midpoint between tip of the acromion and base of neck).

Anodal and cathodal TDCS was delivered with a ramp of 30s up to 2 mA, followed by full intensity stimulation for the duration of the fMRI run, finishing with a ramp down over 1s. Sham TDCS consisted of the ramp stage only. Electrodes had a layer of conductive paste (Ten20, D.O. Weaver, Aurora, CO, USA), which held them in place and reduced impedances. Pre-stimulation impedances were below 3kΩ and maximum impedance during stimulation was 18 kΩ. Heart rate was monitored concurrently in 23 participants using the pulse oximetry of the integrated Siemens Physiological Monitoring Unit. The set up and subsequent signal analysis has been previously described (Violante et al., 2017). There was no effect of stimulation on mean heart rate or its standard deviation.

### 2.6 Diffusion tensor imaging (DTI) acquisition

DTI was performed as the final scan in the session. Diffusion-weighted volumes were acquired using a 64-direction protocol (64 slices, in-plane resolution = 2 × 2 mm, slice thickness = 2 mm, field of view = 25.6 × 25.6 cm, matrix size = 128 × 128, TR = 9500 ms, TE = 103 ms, b-value = 1000  mm^2^ s^−1^). Four non-diffusion weighted images were also acquired (b-value = 0  mm^2^ s^−1^).

### 2.7 Modelling of the current density

A computation model confirmed that the peak electric field strength was over the rIFG. A finite element method (FEM) head model was created using Simnibs (Windhoff et al., 2013; Thielscher et al., 2015). This standard five compartment head model (WM, GM, CSF, skull and skin) was further extended to include neck and shoulder parts. Conductivity values for various tissues were used as in (Opitz et al., 2015). The electrode montage was modelled as described in the experimental section. Simulations of the tDCS electric field were performed using Simnibs v2.0.1.

### 2.8 fMRI preprocessing

Data pre-processing was performed using the FMRI Expert Analysis Tool (FEAT) Version 6.00, from FMRIB's Software Library (FSL (Smith et al., 2004; Jenkinson et al., 2012)). We performed motion correction using MCFLIRT (Jenkinson et al., 2002), removal of low-frequency drifts (high-pass filter of 0.01 Hz), spatial smoothing (Gaussian kernel filter with a full width at half maximum of 6 mm), brain extraction to remove non-brain tissue (BET (Smith, 2002)), and co-registration using FMRIB's Nonlinear Image Registration tool (FNIRT) to register the participant's fMRI volumes to Montreal Neurological Institute (MNI) 152 standard space using the T1-weighted scan as an intermediate.

Single-session ICA was performed for each run using Multivariate Exploratory Linear Optimized Decomposition (MELODIC (Beckmann et al., 2005)). The resulting components were automatically classified into signal and noise using FMRIB's ICA-based Xnoiseifier (FIX (Griffanti et al., 2015; Salimi-khorshidi et al., 2015)). FIX was previously trained in an independent cohort of twenty individuals acquired in the same scanner with the same imaging parameters. Classifications were manually inspected and adjusted when required. Independent components classified as noise were subsequently removed from each voxel's time series.

### 2.9 fMRI task analysis: activation analysis

The fMRI-TDCS SST was analysed with FSL's FMRI Expert Analysis Tool (FEAT) (Smith et al., 2004; Jenkinson et al., 2012). Subject-level general linear models (GLM) included 5 regressors of interest from the task: Go Correct (trials in which correct responses were made to the go signal), Go Incorrect (trials in which incorrect responses were made to the go signal), Stop Correct (trials in which participants successfully withheld a response to the stop signal), Stop Incorrect (trials in which participants pressed a button despite the presentation of a stop signal) and Feedback (presentations of the feedback screen). The GLM design matrix consisted of those regressors of interest, their first temporal derivatives and six movement regressors to account for movement-related noise. The following contrasts of interest were investigated [Stop Correct > Go Correct] and [Stop Correct > Stop Incorrect], and the inverse contrasts were also run.

A higher-level mixed effects (FLAME 1 + 2) analysis of group effects was performed to combine all participants for each stimulation condition (anodal, cathodal and sham). A separate higher-level mixed effects analysis was run to directly compare the stimulation conditions for the contrasts of interest, using the ‘Triple T-test’ GLM set-up within FSL FEAT, allowing direct comparison between stimulation conditions. The final Z statistical images were thresholded using a Gaussian random field-based cluster inference with a height determined by a threshold of z > 3.1 and a corrected cluster significance threshold of p = 0.05.

### 2.10 Diffusion tensor imaging analysis

Diffusion data were preprocessed within FSL and DTITK to build individual Fractional Anisotropy (FA) maps (SI Methods) (Smith, 2002; Zhang et al., 2006, 2010). DTI data were corrected for head motion and eddy current distortions, using linear transformations to register these images to the b = 0 image. A brain mask was generated by brain extracting the b = 0 image (FSL Brain extraction tool (Smith, 2002)). A tensor model was then fitted to the data using FMRIB's Diffusion Toolbox (FDT) in FSL, constrained by the brain mask. Applying this tensor model generated voxelwise individual participant fractional anisotropy (FA) maps. These maps were transformed into 1 mm-resolution standard space using DTI-TK (Zhang et al., 2006). An initial group based template was generated through bootstrapping of the tensor-based maps together with the predefined IXI aging standard template (Zhang et al., 2010). Individual tensor-based images were then registered to the group template using diffeomorphic transformations.

The average FA of the whole white matter skeleton was extracted. An ROI approach was used to assess white matter structural connectivity in the Salience and Default Mode Networks. FA values were extracted from each participant from the following previously described tracts (Bonnelle et al., 2012):

- RAI-dACC/preSMA (right anterior insula to the dorsal anterior cingulate/pre-supplementary motor area) tract – to assess SN structural connectivity from the rAI.
- RIFG-dACC/preSMA (right inferior frontal gyrus to the dorsal anterior cingulate/pre-supplementary motor area) tract – to assess SN structural connectivity from the rIFG, the site of maximal current density.
- mPFC-PCC/PRE (medial prefrontal cortex to posterior cingulate cortex/precuneus) tract – to assess DMN structural connectivity.

We wanted to explore the idea of ultimately using FA as a criteria selecting participants for inclusion in future cognitive TDCS studies. Additionally, the distribution of FA in our group was non-normal, with a rightward skew (Supplementary Data). There are no previous studies suggesting a specific FA value to use as a cut-off. Therefore, participants were stratified into two groups based on whether the tract FA was greater (‘high structural connectivity’) or lower (‘low structural connectivity’) than the median FA within that tract.

## 3 Results

### 3.1 Salience Network stimulation improves response inhibition

**Fig. 2:**
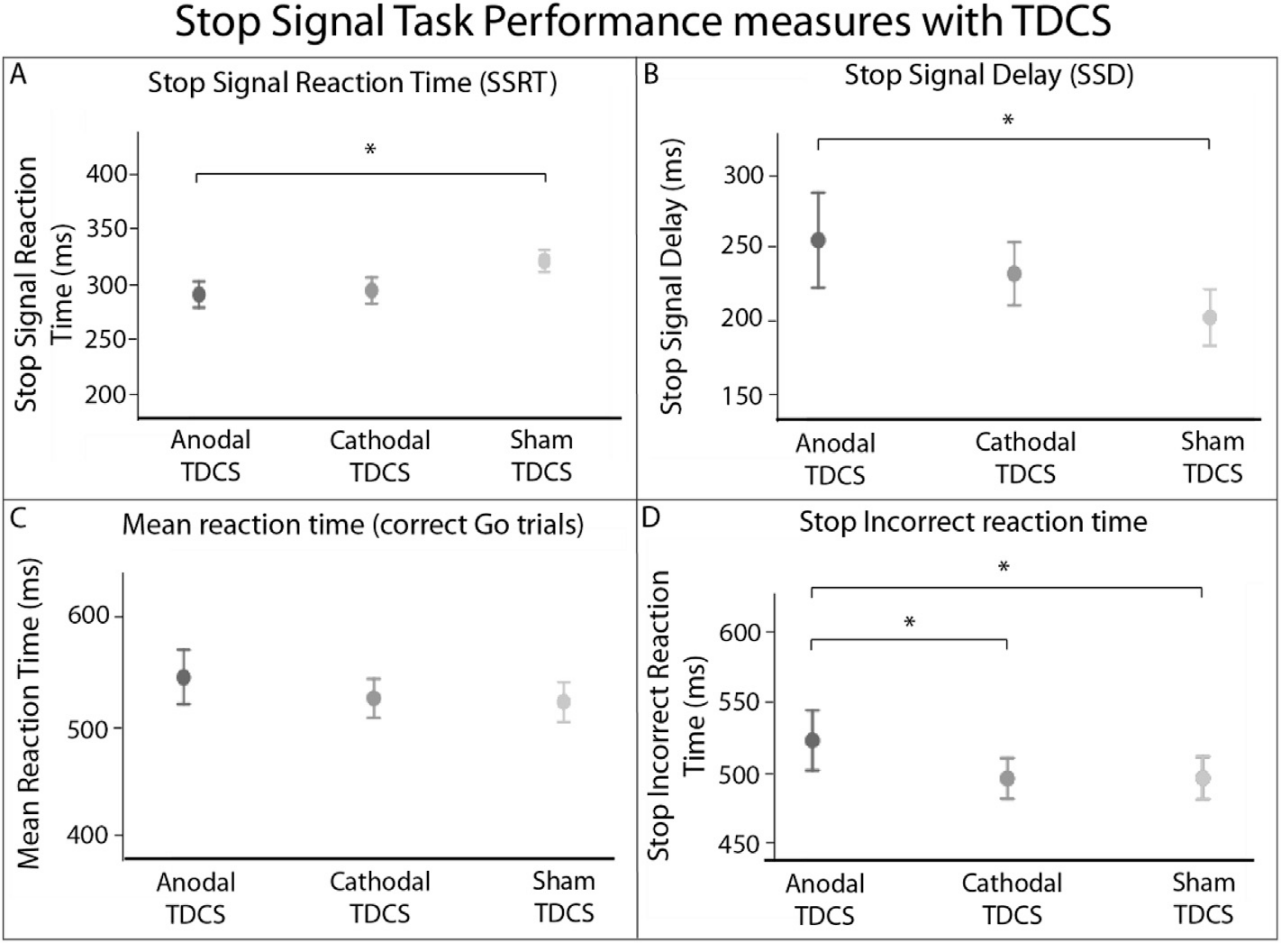
Stop Signal Task Performance measures. (A) Stop Signal Reaction Time, (B) Stop Signal Delay, (C) Reaction Time, and (D) mean Reaction Times for ‘stop’ trials where there is failed response inhibition (‘stop incorrect’ trials) are presented for Anodal, Cathodal and Sham TDCS. Data points are mean values, error bars are SEMs. * denotes p < 0.05.

Anodal TDCS delivered to the right IFG during SST performance caused a significant decrease in the Stop Signal Reaction Time (SSRT) compared with sham TDCS (t (23) = 2.17, p = 0.04) (Table 1, Fig. 2A). This effect was not seen for cathodal TDCS, although it approached significance (t (23) = 1.87, p = 0.07). There was no significant difference in SSRT between anodal and cathodal TDCS (t (23) = 0.25, p = 0.81). The SSRT is a composite measure (mean reaction time – stop signal delay). Anodal TDCS improved the stop signal delay (SSD), indicative of improved response inhibition, rather than a change in the mean reaction time (Fig. 2B and C). This indicates that the change in SSRT reflects improved response inhibition rather than motor slowing. In addition, on ‘Stop’ trials where participants failed to inhibit their response (Stop Incorrect trials), anodal TDCS also delayed the time taken to produce an incorrect response, compared with both sham (t (23) = 2.32,p = 0.03) and cathodal (t (23) = 2.15, p = 0.04) TDCS (Fig. 2d). There were no effects of TDCS on other behavioural measures (Table 1).

**Table 1:** Behavioural measures for the Stop Signal Task. Figures shown are mean values ± standard deviation. Abbreviations: RT = reaction time, ms = milliseconds.

	Anodal	Cathodal	Sham
Stop Signal RT (ms)	291.3 ± 58.4	295.1 ± 58.6	321.7 ± 48
Stop Signal Delay (ms)	253.7 ± 159.3	230.8 ± 105.7	200.8 ± 94.9
Incorrect stop RT (ms)	523.0 ± 104.7	495.8 ± 71.5	496.1 ± 75.1
Mean RT (ms)	545.1 ± 121.1	525.9 ± 87.6	522.6 ± 89.4
Go accuracy (%)	95.1 ± 7.2	96.1 ± 7.4	95.0 ± 7.6
Stop accuracy (%)	48.5 ± 4.9	48.3 ± 4.0	49.8 ± 3.7
Negative feedback	13.1 ± 10.6	11.4 ± 7.5	11.9 ± 9.5

Salience Network structural connectivity influences behavioural and physiological responses to Anodal but not Cathodal TDCS.

**Fig. 3:**
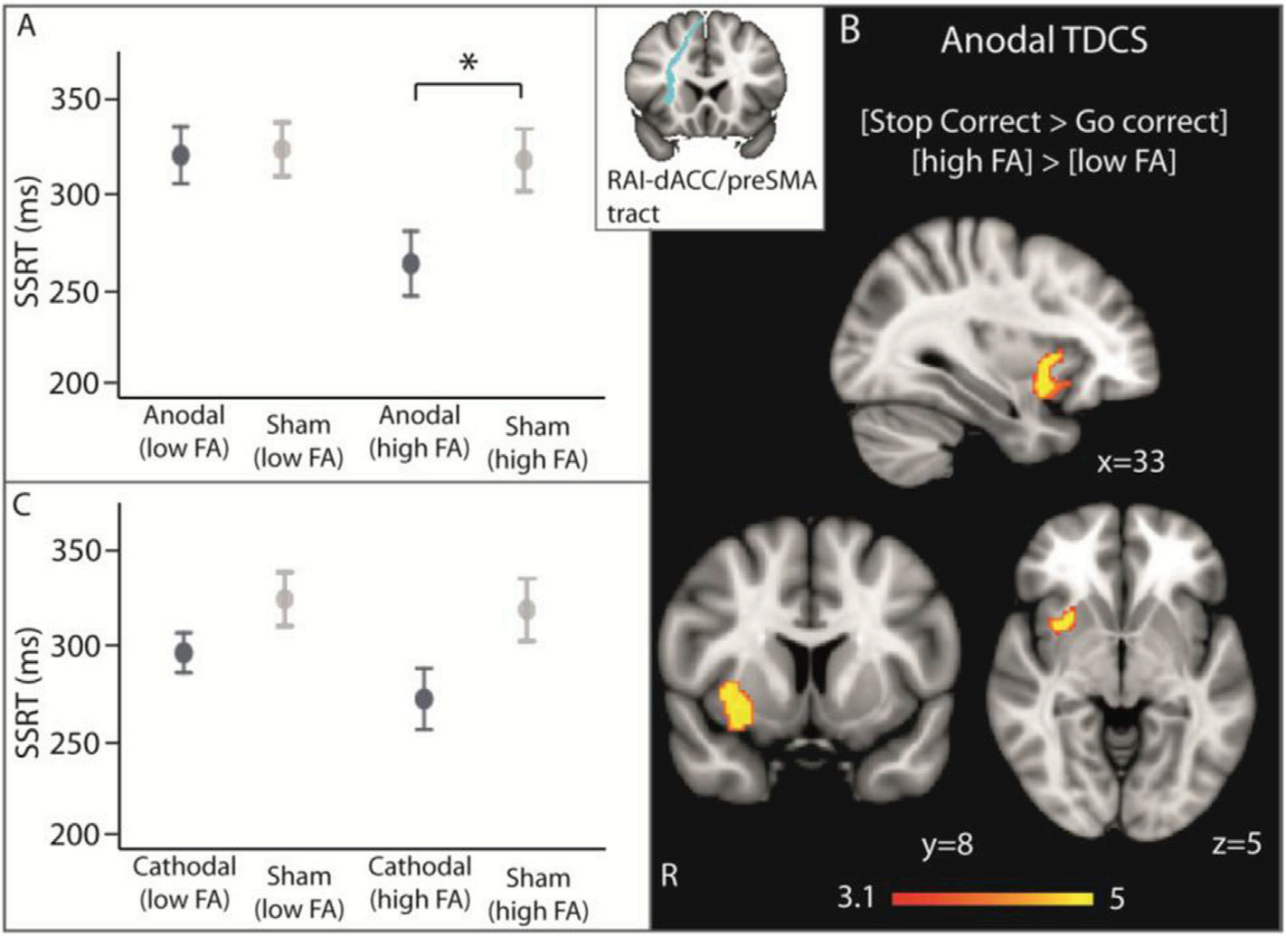
The effect of rAI-dACC/preSMA tract FA on response to TDCS. (A) SSRT performance under anodal TDCS, (B) brain activation under anodal TDCS and (C) SSRT performance under cathodal TDCS were compared between participants with high and participants with low rAI-dACC/preSMA FA. Inset shows the white matter tract mask used to extract FA values. Error bars are S.E.M. *indicates p < 0.05, Bonferroni corrected for multiple comparison. FMRI results are superimposed on the MNI152 1 mm brain template. Cluster corrected z = 3.1, p < 0.05.

We have previously shown that damage to the structural connectivity within the SN strongly predicts how efficiently network activity is co-ordinated during response inhibition (Bonnelle et al., 2012). Therefore, we tested whether stratifying participants based on fractional anisotropy (FA) of the SN influenced the response to TDCS. FA of the tract connecting the rAI and the dACC/preSMA nodes of the SN strongly predicted response to anodal TDCS. Participants with high FA within the rAI-dACC/preSMA tract had significantly improved SST performance under anodal TDCS compared with sham TDCS (t (10) = -3.97 p = 0.0026). Conversely, those with low FA within the SN showed no improvement in performance under anodal TDCS compared with sham TDCS ((t (10) = 0.18 p = 0.88) (Fig. 3a). The FA of this tract was negatively correlated with the SSRT under anodal TDCS, reflecting better SST performance with higher FA, though this correlation was not significant (r = −0.3, p = 0.17) (Supplementary Data).

**Fig. 4:**
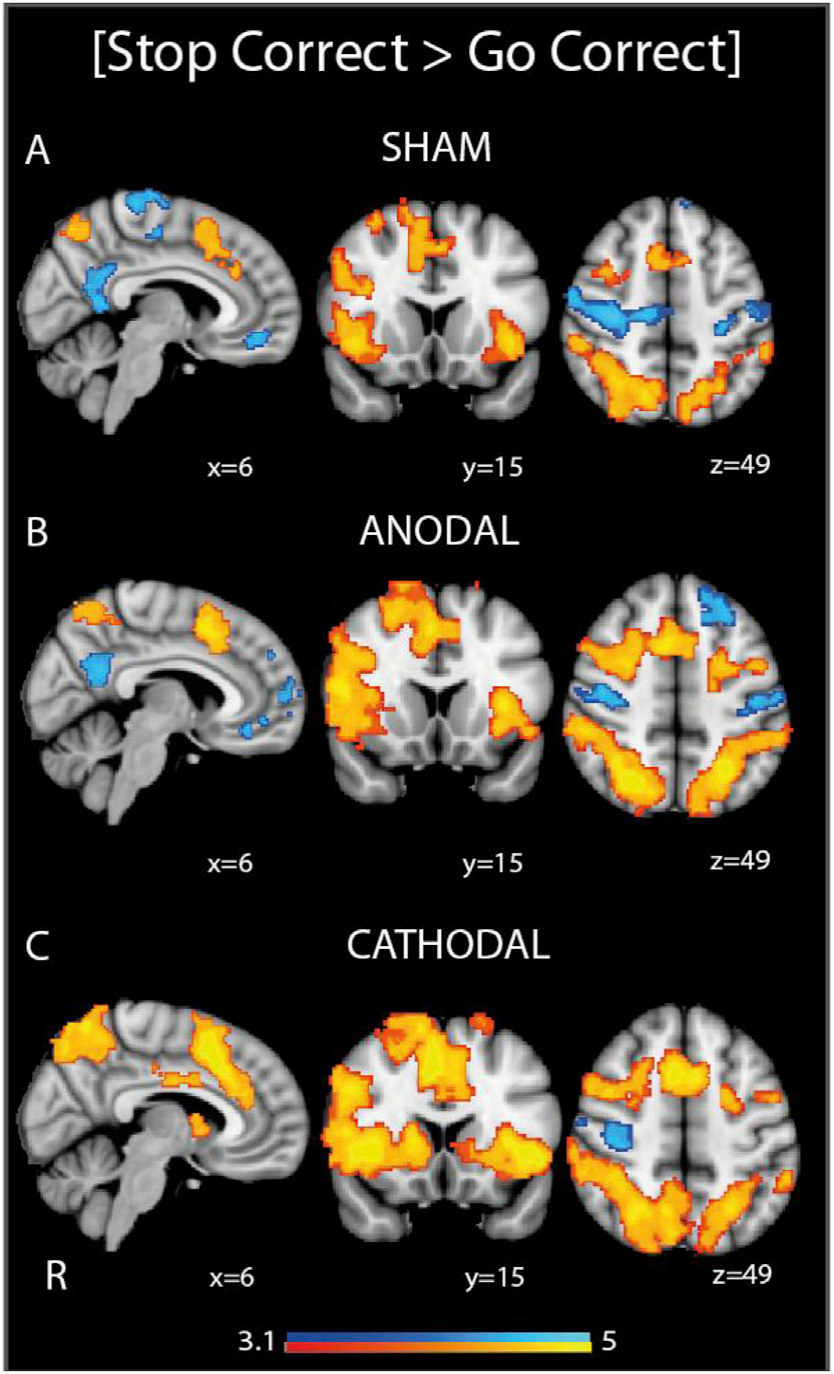
Effect of TDCS on network activity. Overlay of areas of brain activation (warm colours) and deactivation (cool colours) under sham, anodal and cathodal TDCS, during successful response inhibition (successfully withholding a button press when a ‘stop’ signal is presented, compared with correctly responding to arrows without a ‘stop’ signal). Results are superimposed on the MNI152 1 mm brain template. Cluster corrected z = 3.1, p < 0.05.

The FA of the SN also influenced the effect of anodal TDCS on brain activity during response inhibition. The contrast [Stop Correct > Go Correct] denotes successfully witholding button presses to presentation of the ‘stop’ signals, compared with correctly responding with button presses to the arrows. This contrast demonstrates brain regions involved in detecting and attending to the ‘stop’ signal, as well as inhibiting the automatic ‘go’ response. In line with previous literature, this contrast showed increased activity within the Salience Network (SN) and bilateral superior parietal areas, along with deactivation in the Default Mode Network (DMN) and primary sensorimotor regions (Fig. 4a).

The broad patterns of SN and frontoparietal activation and DMN deactivation were preserved with the addition of anodal or cathodal TDCS (Fig. 4). At a group level anodal TDCS did not significantly change the pattern of brain responses for [Stop Correct > Go Correct] (Fig. 4B). However, anodal TDCS produced greater activation in the right anterior insula, an area already activated during this contrast, in participants with ‘high’ FA in the rAI-dACC/preSMA tract than in those with low FA (Fig. 3B). The FA of this tract was significantly positively correlated with BOLD response of the right anterior insula, reflecting a greater BOLD response with higher FA (r = 0.53, p = 0.01) (Supplementary Data). This effect was specific to successful response inhibition, as there were no activation differences for the contrast [Stop Incorrect > Go Correct], which additionally controls for the effect of ‘stop’ signal presentation, between those with ‘high’ and ‘low’ tract FA.

The influence of rAI-dACC/preSMA tract FA on the physiological and behavioural response to TDCS appeared to be specific to response to anodal TDCS. There were no differences in behaviour or brain activation between participants with ‘low’ or ‘high’ rAI-dACC/preSMA tract FA under cathodal (Fig. 3C), or sham tDCS. Additionally, these effects were also specific to FA of the rAI-dACC/preSMA tract of the Salience Network. There were no physiological or behavioural differences between participants with ‘low’ or ‘high’ whole brain FA, the FA of the SN tract linking the rIFG to the dACC/preSMA or the tracts linking the ventromedial prefrontal cortex with the posterior cingulate cortex, the two main nodes of the DMN.

### 3.2 Cathodal TDCS produces subcortical activation during response inhibition

**Fig. 5:**
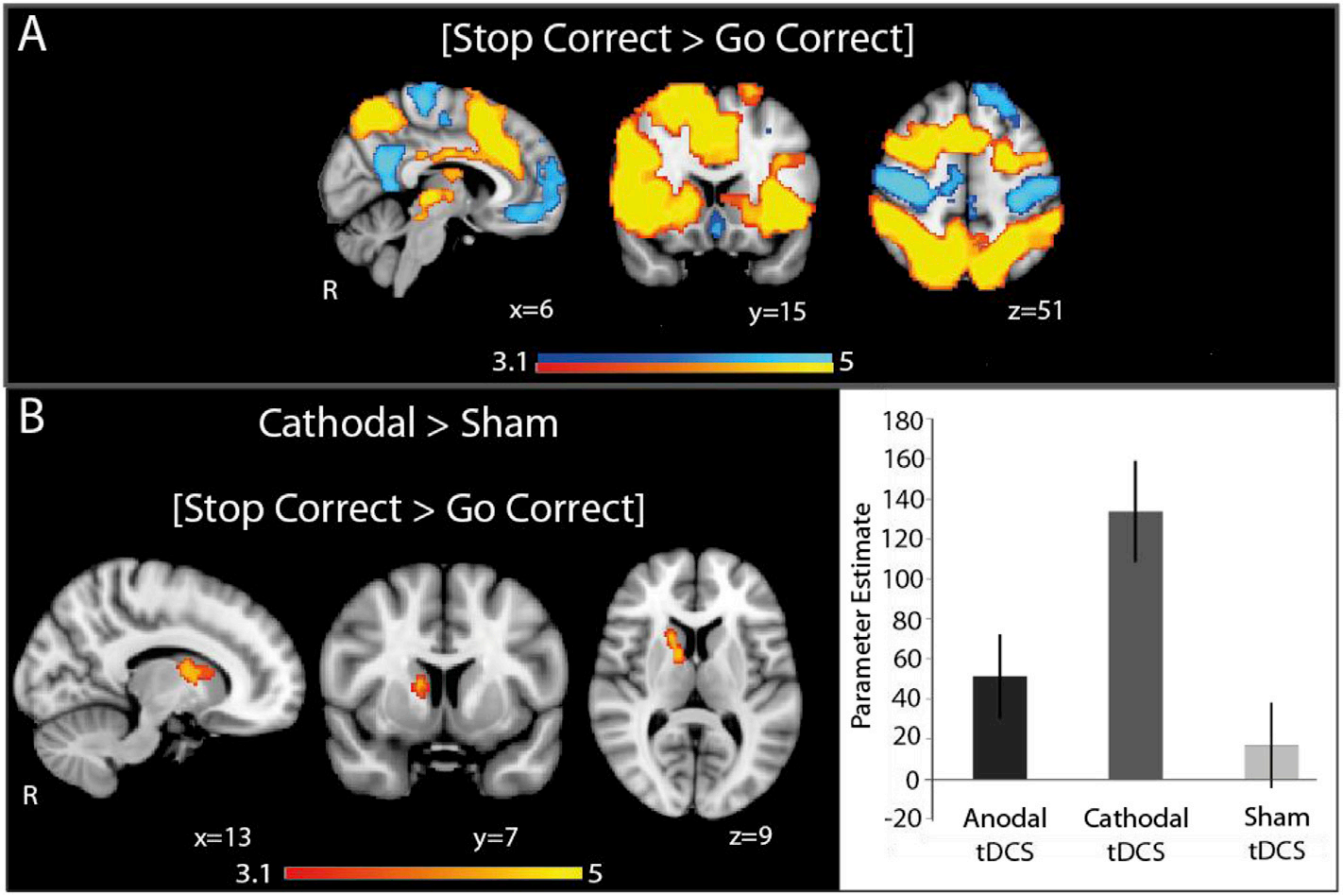
Effect of cathodal TDCS on brain BOLD response. (A) Overlay of areas of brain activation (warm colours) and deactivation (cool colours), across all stimulation conditions, for [Stop Correct > Go Correct] and [Stop Correct > Stop Incorrect]. (B) Overlay of areas where brain activation (warm colours) is greater under cathodal TDCS than sham TDCS during successful stopping. Results are superimposed on the MNI152 1 mm brain template. Cluster corrected z = 3.1, p < 0.05. Accompanying bar charts show mean activation within the activated areas, and demonstrate the change in Parameter Estimate with each type of stimulation. Error bars denote S.E.M.

There was a group-level difference in brain activity with cathodal TDCS compared to sham TDCS (Fig. 4, Fig. 5). During response inhibition, cathodal TDCS produced increased activation within the right caudate, compared with sham (Fig. 5b). There were no group level differences in activity patterns between anodal and cathodal TDCS, nor were there any correlations between stimulation-induced changes in BOLD activity and behaviour.

### 3.3 Current density distribution

Current density modelling confirmed that the peak of the electric field distribution over the cortical surface was centred over the right inferior frontal gyrus (Fig. 1c). Current spread into underlying structures, including the right frontal operculum and right anterior insula. High electric field strengths were also found within SN tracts connecting these areas to the dACC/preSMA. A more remote peak of current was seen within the corpus callosum.

### 3.4 Control analysis: perception of TDCS

Participants were asked whether they thought they had real or sham TDCS after the end of each run. Their accuracy rates were consistent with chance (50% after anodal, 55.7% after cathodal and 46.1% after sham). There were no differences between stimulation conditions in ratings of perceived sensations during the run.

## 4 Discussion

Our results support a causal influence of the Salience Network (SN) in cognitive control. We show that anodal TDCS applied to the right inferior frontal gyrus/anterior insula (rIFG/AI) node of the SN improves response inhibition, as measured by the Stop Signal Task (SST). We targeted this region aiming to influence the function of the Salience Network (SN), since dynamic changes in rAI activity during SST performance causally influences other parts of the SN and anti-correlated activity within the Default Mode Network (DMN) (Sridharan et al., 2008; Menon and Uddin, 2010; Dosenbach et al., 2006). A previous study found that higher fractional anisotropy (FA) within the rIFG and preSMA of children was correlated with better SST performance, and we have previously found that structural damage of the rAI-dACC/preSMA tract of the SN is a strong predictor of the network interactions associated with worse response inhibition (Bonnelle et al., 2012; Jilka et al., 2014). We now extend these observations by showing how SN structure also influences behavioural and physiological responses to brain stimulation over one node of the SN. High FA within this tract was associated with both a behavioural and physiological response to TDCS, effects not observed with cathodal TDCS or for other key white matter tracts.

Two previous stroke studies showed a relationship between white matter tract FA and the behavioural response to contralesional cathodal TDCS (Rosso et al., 2014), (Bradnam et al., 2012). A recent healthy participants study found a correlation between the analgesic effects of anodal TDCS to the left dorsolateral prefrontal cortex and the structural connectivity of its thalamic connections (Lin et al., 2017). We now show a clear relationship between rAI-dACC/preSMA tract FA and the cognitive and physiological effects of TDCS to the SN.

Several possibilities may explain why individual differences in white matter FA within a network might influence its response to stimulation. One possibility is that higher FA represents higher structural connectivity between nodes of a network, allowing a stimulated cortical region to exert stronger effects across the network. Individual variability in structural connectivity influences network interactions (Boorman et al., 2007; Honey et al., 2009; Horn et al., 2014). Thus, variation in the structural connectivity from a stimulated region might influence the network's response during task performance, and therefore the behavioural effects of this stimulation. This may explain why we did not find an influence of DMN structural connectivity on TDCS effects, because key DMN nodes were not within the electrical field of stimulation.

Another possibility is that behavioural effects of TDCS are at least partially mediated by axonal polarisation. TDCS studies to date have generally targeted cortical structures in the belief that stimulation effects mainly result from changes in soma polarisation. However, recent evidence shows that polarisation effects can be stronger at axon terminals than soma (Chakraborty et al., 2017). Our modelling also predicts peaks of electric field strength in the SN white matter tracts. Hence, the effect of TDCS might result from direct polarisation of axonal membranes within white matter tracts connecting the SN nodes. Amongst other things, white matter FA can reflect the mix of fibre orientations within a tract, in part quantifying the variability of axonal orientation (Winston, 2012). Axonal orientation has been shown to particularly influence the polarising effect of TDCS (Arlotti et al., 2012; Kabakov et al., 2012). Therefore, interindividual variability in tract FA may lead to variation in the sensitivity of an individual's tract to the polarising effects of TDCS.

A third possibility is that the current density distribution is modulated by variability in the FA of underlying tracts, with high FA perhaps resulting in more focussed cortical stimulation. Computational modelling studies suggest that the physiological effects of TDCS are likely to interact with the properties of white matter tracts underlying stimulated cortex, albeit to a small extent (Metwally et al., 2012; Shahid et al., 2012, 2014). This is unlikely to be a major factor in explaining our findings since we show an influence of FA variability of the rAI-dACC/preSMA tract specifically, but not of the overlying rIFG connections, where modelling predicts high electric field strengths.

The particular anatomy of the rAI and its projections may make it especially susceptible to the effect of TDCS. The rAI of great apes and humans contain an unusual set of large bipolar projection neurons called Von Economo neurons (VENs). They are thought to be specialised for sending information rapidly, which may have evolved to facilitate the fast signalling required for rapid behavioural changes in response to environmental changes (von Economo, 1926; Seeley et al., 2012; Allman et al., 2011). Large bipolar neurons are more sensitive to soma polarisation, possibly because they have a greater surface area of membrane on which the current can act (Arlotti et al., 2012; Radman et al., 2009). The large size and relatively simple dendritic structure may make VENs, and thus the rAI, particularly sensitive to the effects of TDCS. An extension of our study would be to investigate whether focused stimulation of the dACC/preSMA, where VENs are also found, replicates our findings.

The physiological response to anodal TDCS was also influenced by the FA of the rAI-dACC/preSMA tract. Participants in the ‘high’ FA group showed greater activation within the rAI, which is particularly important for switching behaviour in general (Sridharan et al., 2008). This suggests that the improved task performance in ‘high’ FA participants with anodal TDCS may be mediated by more general processes involved in cognitive control, rather than specific motor inhibition mechanisms. Our findings raise the interesting question of whether there is a FA ‘threshold’ required for TDCS effectiveness. This is particularly important as patients with low FA due to white matter injury may respond very differently to stimulation compared to controls or patients with preserved white matter FA. The influence of white matter structure on response to TDCS should be further investigated in different patient groups, particularly to assess whether sub-groups of patients can be defined on the basis of FA in key stimulated tracts.

Our results also highlight that TDCS applied to a single key node can produce effects in remote but connected brain regions. Cathodal TDCS increased caudate activation even though the caudate is not predicted to experience high electrical field strengths. The rIFG and caudate are connected by white matter tracts, and TDCS of the motor cortex can modulate functional connectivity between motor association cortices and subcortical structures, including the caudate (Polanía et al., 2012; Catani et al., 2011). It is not completely clear how the physiological changes seen with cathodal TDCS relate to behaviour since there was no direct correlation between the caudate activation and behavioural effects induced by cathodal TDCS. However, subcortical structures are also known to make important contributions to cognitive control, and further studies should investigate the extent to which modulation of fronto-subcortical circuits by cathodal TDCS can modulate cognitive control (Aron and Poldrack, 2006; Jahfari et al., 2012; Jahanshahi et al., 2015).

Our study supports the view that polarity, as applied to cognitive functions, is more complex than an anodal-excitatory/cathodal-inhibitory dichotomy. There were no significant behavioural differences between anodal and cathodal TDCS, though there were distinct patterns of brain network activity with anodal and cathodal TDCS. The behavioural and physiological effects of anodal TDCS were dependent on SN tract FA, which is not observed for cathodal TDCS. Conversely, cathodal TDCS increased subcortical activation during response inhibition, which anodal TDCS did not. Whilst our current study raises many interesting questions about the relative effects of polarity, we can only speculate about the mechanisms behind these observed differences in polarity effects. In vitro studies suggest that anodal and cathodal TDCS have opposing effects on neuronal excitability and inhibitory/excitatory balance (Stagg and Nitsche, 2013), but how this translates to activity of neuronal circuits, BOLD activity and then cognitive performance is far less certain. The direction of current flow and the orientation of neuronal processes within the electric field have both been shown to be important determinants of soma and axonal polarisation (Arlotti et al., 2012; Rahman et al., 2013). Anodal and cathodal TDCS represent different current flow directions. A speculative reason for polarity-dependent effects may be the presence of neuronal subpopulations within the rIFG. For example, if a particular subpopulation, with projections to the caudate, had neuronal orientations that made them more responsive to the cathodal current direction, it might explain why only cathodal TDCS produced substantial subcortical activation. Much more work is required to fully investigate the mechanisms of polarity-dependent network effects, and their relationship to behavioural effects.

It is unlikely that the behavioural effects of TDCS result from non-specific effects. First, post-stimulation questionnaires indicated that participants were unaware of whether they received real or sham TDCS, nor were there differences between stimulation conditions on perception of possible TDCS-related sensations. Secondly, the polarity-specific effects on brain activation and on the influence of SN tract structure cannot be wholly explained by non-specific effects.

The main limitation of our study is that we only investigated response inhibition with the SST, so cannot comment on whether SN TDCS could modulate other aspects of cognition, or whether rAI-dACC/preSMA tract FA would be a relevant differentiator of TDCS responders. Given the importance of the SN in coordinating cognitive network activity and role in many different cognitive functions, we would hypothesis that our findings could be replicated for other cognitive tasks. We also did not assess whether targeting the other main SN node, the dACC/pre-SMA, would have a similar effect. One potential limitation relates to our sample size. We clearly show an effect of rAI-dACC/pre-SMA tract FA on the behavioural and functional imaging effects of anodal TDCS. These results survive stringent multiple comparison correction. More generally, our sample is comparable or bigger than other studies investigating the fMRI imaging correlates of stimulation (Antal et al., 2011; Peña-Gómez et al., 2012; Kasahara et al., 2013; Polanía et al., 2011; Sehm et al., 2012b). Although anodal and cathodal TDCS produced distinct patterns of imaging results when related to sham, we did not show clear differences between anodal and cathodal stimulation in direct behavioural or imaging contrasts. This null result may reflect a lack of power for this particular analysis, and further work will be necessary to clarify this. Our findings highlight the need for further research, ideally using multimodal approaches that enable simultaneous investigation of multiple levels of polarity effects such as neuronal circuit physiology and neurotransmitter changes, to fully elucidate the mechanisms for differences in polarity effects in the cognitive domain.

## 5 Conclusions

Our study demonstrates that anodal TDCS applied to the SN can improve response inhibition and alter patterns of SN activation, an effect dependent on the structural connectivity of the SN. We also show that SN stimulation with cathodal TDCS can remotely modulate DMN functional connectivity. These results extend our understanding of the role of the SN in cognitive control, support SN stimulation as an attractive option for cognitive enhancement, and highlight the importance of network structural connectivity as a modulator of the effects of stimulation.

## Author contributions

LML, IV and DJS conceived the study and designed the experiments. DC advised on experimental design. LML and IV acquired the data. AO conducted the current density modelling. RL, AH and DM advised on data analysis. LML wrote the first draft of the manuscript. All authors reviewed and edited the final manuscript. DJS supervised the study.

## Declaration of interests

The authors declare no competing interests.
