# Supplementary material for: Cognitive enhancement with Salience Network electrical stimulation is influenced by network structural connectivity
Source: Neuroimage. 2019 Jan 15;185:425–33. doi: 10.1016/j.neuroimage.2018.10.069 (PMC6299257; doi:10.1016/j.neuroimage.2018.10.069)

**Supplementary Data**

Figure 1:

Distribution of FA within the rAI-dACC/preSMA white matter tract


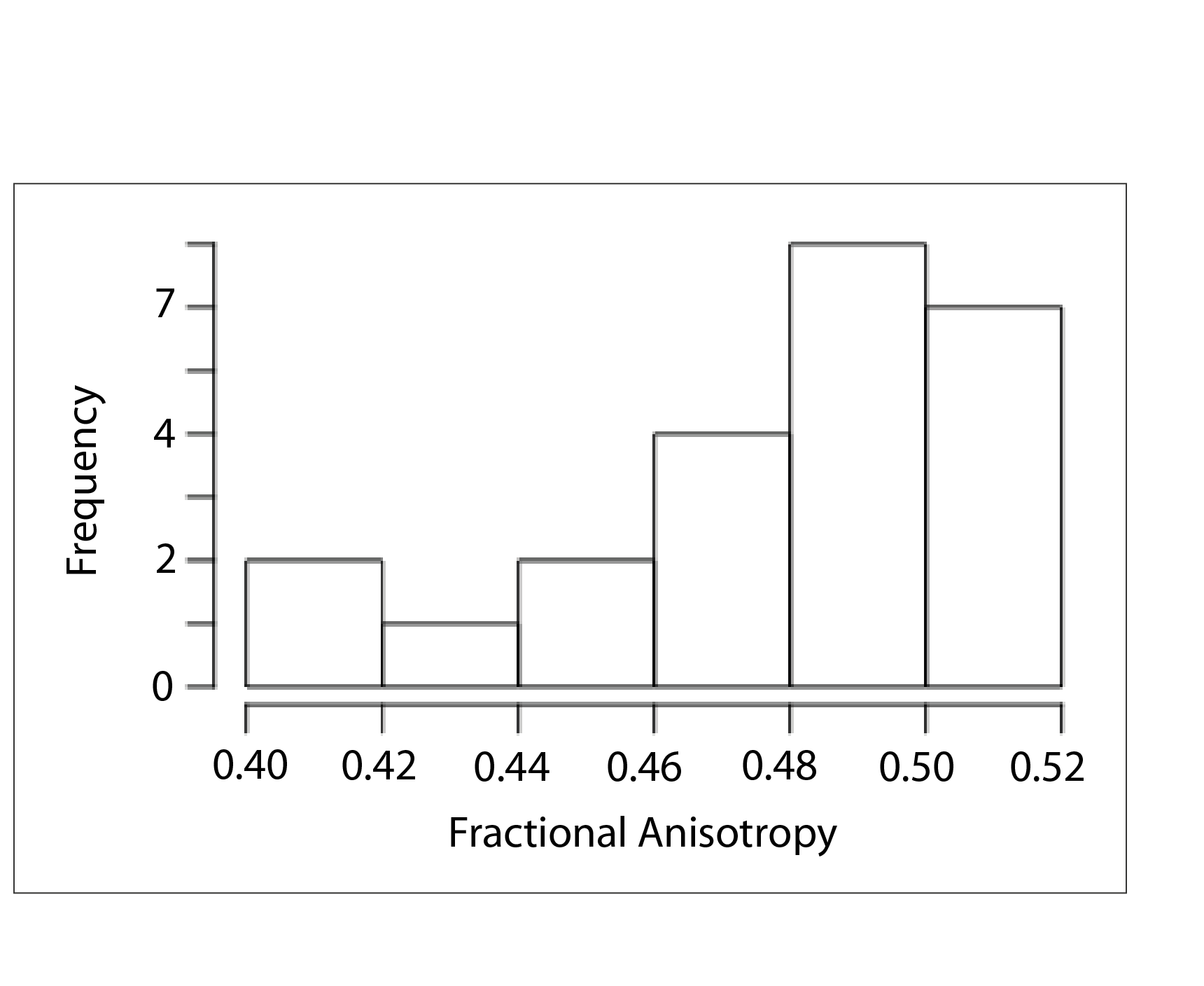


Figure 2:

Relationship between rAI-dACC/preSMA tract FA and rAI BOLD activity under anodal TDCS (r=0.53, p=0.01)


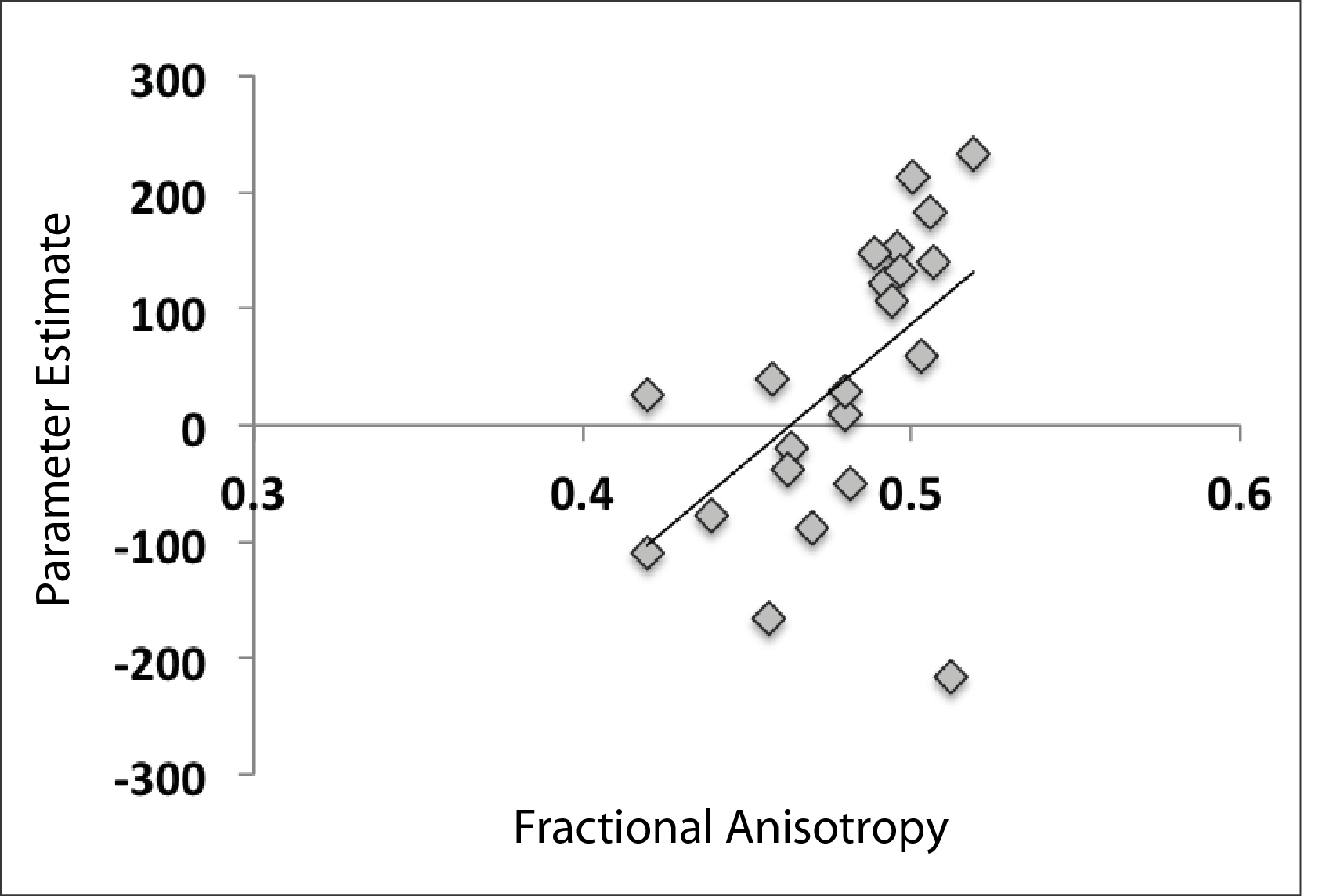


Figure 3:

Relationship between rAI-ACC/preSMA tract FA and SSRT under anodal TDCS (r=-0.30, p=0.17)


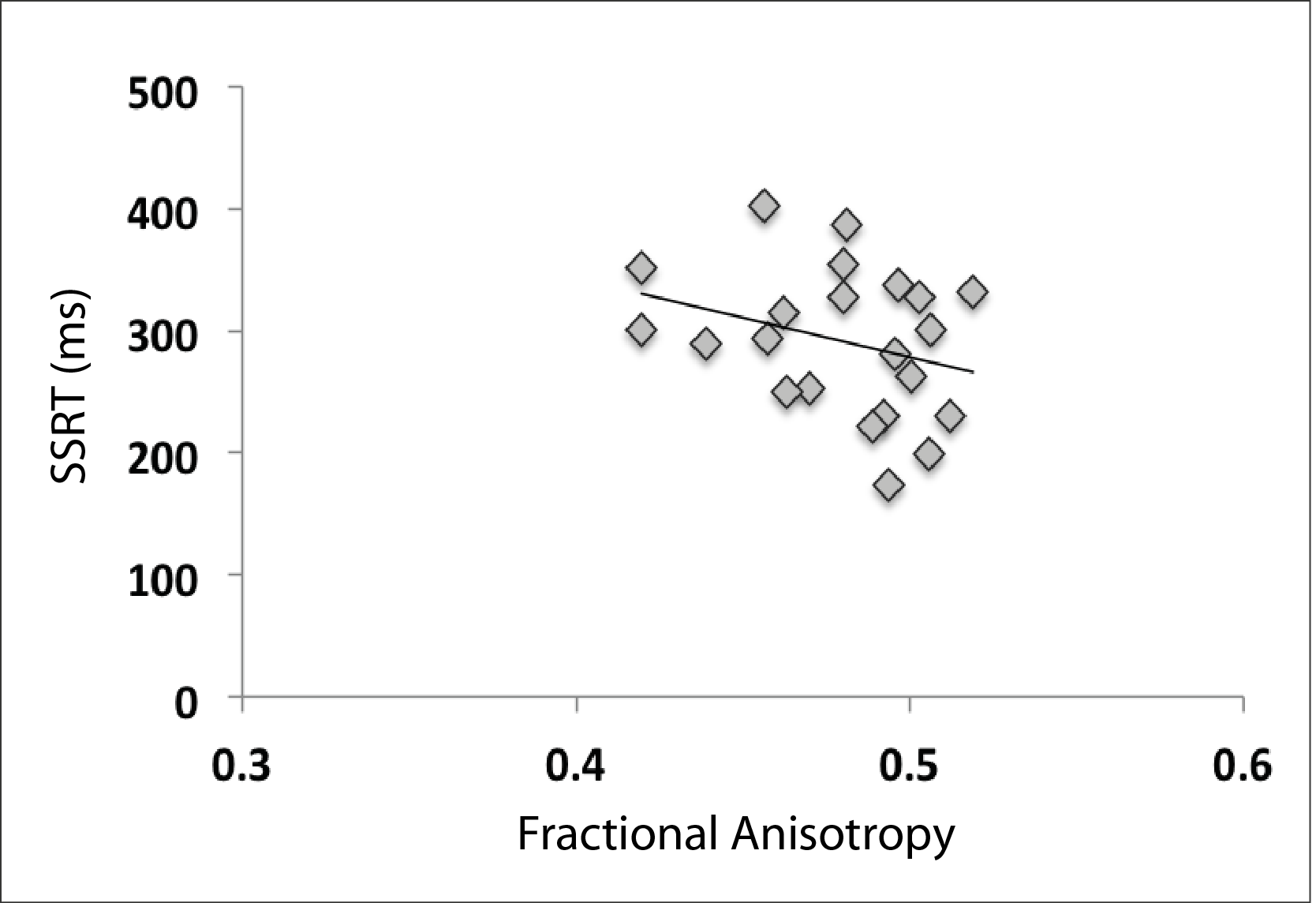

Supplement: Multimedia component 1 [file mmc1.docx]
